# Coupling of Cellular Processes and Their Coordinated Oscillations under Continuous Light in *Cyanothece* sp. ATCC 51142, a Diazotrophic Unicellular Cyanobacterium

**DOI:** 10.1371/journal.pone.0125148

**Published:** 2015-05-14

**Authors:** S. Krishnakumar, Sandeep B. Gaudana, Nguyen X. Vinh, Ganesh A. Viswanathan, Madhu Chetty, Pramod P. Wangikar

**Affiliations:** 1 Department of Chemical Engineering, Indian Institute of Technology Bombay, Powai, Mumbai, India; 2 DBT Pan IIT Center for Bioenergy Indian Institute of Technology Bombay, Powai, Mumbai, India; 3 Wadhwani Research Center for Bioengineering, Indian Institute of Technology Bombay, Powai, Mumbai, India; 4 School of Information Technology, Federation University Australia, Gippsland Campus, Victoria, 3841, Australia; University of Wisconsin Milwaukee, UNITED STATES

## Abstract

Unicellular diazotrophic cyanobacteria such as *Cyanothece* sp. ATCC 51142 (henceforth *Cyanothece*), temporally separate the oxygen sensitive nitrogen fixation from oxygen evolving photosynthesis not only under diurnal cycles (LD) but also in continuous light (LL). However, recent reports demonstrate that the oscillations in LL occur with a shorter cycle time of ~11 h. We find that indeed, majority of the genes oscillate in LL with this cycle time. Genes that are upregulated at a particular time of day under diurnal cycle also get upregulated at an equivalent metabolic phase under LL suggesting tight coupling of various cellular events with each other and with the cell’s metabolic status. A number of metabolic processes get upregulated in a coordinated fashion during the respiratory phase under LL including glycogen degradation, glycolysis, oxidative pentose phosphate pathway, and tricarboxylic acid cycle. These precede nitrogen fixation apparently to ensure sufficient energy and anoxic environment needed for the nitrogenase enzyme. Photosynthetic phase sees upregulation of photosystem II, carbonate transport, carbon concentrating mechanism, RuBisCO, glycogen synthesis and light harvesting antenna pigment biosynthesis. In *Synechococcus elongates* PCC 7942, a non-nitrogen fixing cyanobacteria, expression of a relatively smaller fraction of genes oscillates under LL condition with the major periodicity being 24 h. In contrast, the entire cellular machinery of *Cyanothece* orchestrates coordinated oscillation in anticipation of the ensuing metabolic phase in both LD and LL. These results may have important implications in understanding the timing of various cellular events and in engineering cyanobacteria for biofuel production.

## Introduction

Cyanobacteria are being keenly explored for carbon dioxide capture and conversion to biomass and biofuels [1,2]. Cyanobacteria, a group of prokaryotes, perform oxygenic photosynthesis and are responsible for a significant fraction of primary production on earth. The fixed organic carbon can be utilized directly or processed into high value commodities including biofuels. Some cyanobacteria also fix nitrogen, contribute significantly to the global nitrogen cycle [3] and may play an important role in the nitrogenase mediated production of hydrogen [4]. The nitrogenase enzyme complex is sensitive to molecular oxygen and therefore, cyanobacteria are remarkable for their ability to perform oxygenic photosynthesis and nitrogen fixation within a single cell. Unicellular nitrogen fixing cyanobacteria such as *Cyanothece* sp. temporally separate these seemingly incompatible metabolic pathways [5,6] while heterocyst forming cyanobacteria such as *Nostoc* sp. employ spatial separation to protect the nitrogenase enzyme from molecular oxygen [7].

Several studies have demonstrated that the global gene expression in cyanobacteria is under the control of an internal circadian clock that pre-empts the need to sense light or time of the day in real time [8].This provides an advantage to the cells as they are able to get ready for the ensuing day or night. While the clock components and the mechanism of circadian control are most well enumerated in a model cyanobacterium *Synechococcus elongatus* PCC 7942 (henceforth *Synechococcus* 7942) [9], all sequenced cyanobacteria contain majority of the key clock components [10]. The circadian control is exerted through a core molecular clock, input pathways that sense environmental cues and output pathways that transmit signal to the functional genes. The core clock is comprised of three proteins: KaiA, KaiB and KaiC with KaiC as the central oscillator that undergoes phosphorylation-dephosphorylation cycles of 24 h period [11,12]. The core clock receives cues from the environment through the components of input pathway such as CikA and RpaA and passes on signal to the output pathway components such as PatB, NtcA and SasA [13]. The input to the clock is most likely received through physiological parameters such as intracellular redox state [14] or the ATP to ADP ratio [15,16].

In recent years, *Cyanothece* sp. ATCC 51142 (henceforth *Cyanothece* 51142), a unicellular diazotroph, is being explored as another model organism for studies on the circadian rhythm [17–20]. *Cyanothece* 51142 is reported to produce highest amount of hydrogen amongst all the studied wild type cyanobacterial strains. Importantly, the strain shows robust oscillations in metabolism even under continuous light. The oscillations can be observed as rhythmic buildup and depletion of intracellular glycogen as well as in real time with exhaust gas CO_2_ and O_2_ analysis [21,22]. This enables sampling of the culture under specific metabolic phases and subsequent correlation of the gene expression results with the physiology of the organism. On the contrary, *Synechococcus elongatus* PCC 7942 (henceforth *Synechococcus* 7942) does not show any appreciable oscillations in metabolism under LL despite significant and observable oscillations in gene expression [23,24].

In a previous study, the rhythmicity of gene expression of *Cyanothece* 51142 under LL may not have been fully captured possibly because the study was limited to the first 24 h of LL [25]. We have recently shown that although the culture shows a cycle time of ~11 h under LL, it takes ~20 h for the new rhythm to set in after switching to LL [18]. To the best of our knowledge, the present study is the first one that correlates the oscillations in global gene expression to those in metabolism in cyanobacteria in continuous light. We observe that genes associated with a large number of diverse cellular functions including but not restricted to central metabolism and circadian rhythm oscillate with a period of ~11 h under LL. Starting with a formal treatment for periodicity analysis, we investigate into the temporal juxtaposition of the various cellular events in a shortened “day” of ~11 h in *Cyanothece* 51142. Our analysis points to an interesting possibility that the metabolic state of the cell drives the “circadian rhythm” rather than the opposite.

## Materials and Methods

### Culture growth

Axenic cultures of *Cyanothece* 51142 were cultivated in ASP2 medium without nitrate [6] in air-sparged and stirred bioreactor with external illumination of 230 μmol photons, as described earlier [18]. The organism was grown under highly turbulent conditions, equivalent to 30000 Reynolds number, to simulate flashing light effect [22]. The culture was entrained under light/dark cycles (12 h /12 h) (LD) for 4 days and then released into continuous light (LL). The growth of the organism was monitored by measuring the optical density at 730 nm (OD_730nm_).

### RNA extraction and microarray

Samples were drawn to extract RNA for microarray analysis such that the product of volume and OD730nm was 50.0. The same RNA samples were also used for Real-time PCR analysis for validation of microarray results [18]. Nine samples were drawn from two consecutive cycles under LL (S1 Fig). Microarray analysis was performed at Mogene (St. Louis, USA) as described earlier [19]. Briefly, total RNA from individual samples and an equi-molar mixture of all samples as control were labeled with Cy3 and Cy5, respectively. Unbound dyes were removed using RNA clean kit (Zymo Research, Irvine, CA). Two biological replicates, two technical replicates and a dye swap were analyzed for each sample from individual time points. The microarray dataset submitted to Gene Expression Omnibus (GEO) database, accession no. GSE65429.

### Models for identifying gene periodicity

We employ two complementary models to detect gene periodicity. First, we fit a single sinusoidal component to expression profile of each gene [26]. This model can be used for exploratory investigation into major periodicities present in the data. Subsequently, we employ a more rigorous statistical test for periodicity based on the Fourier score [27]. This test requires that a suspected periodicity T (or a set of periodicities) be specified. To ensure statistical rigor, we select genes that satisfy both conditions.

### Sinusoidal model for Periodicity

As in [26], the gene expression dynamics can be modeled by employing a sinusoidal component together with a linear term (Eq (1)).

g(t)=a+bt+Asin(2πTt+ϕ)+error(t) (1)

where (*A*, *T*, *ϕ*) are the amplitude, period and phase of the sinusoidal component, while the (*a* + *b t*) term reflects the linear trend of expression level and may account for temporal drift in the data.

Let (*t*_*i*_, *g*_*obs*_ (*t*_*i*_)), *i* = *1…n* be the actual (time, observed measurement) pairs for the n time points. We first remove the linear trend via linear regression, with a and b estimated as:

$$\begin{array}{c} a=\frac{\sum g_{obs}(t_{i})-b\sum t_{i}}{N},\\ b=\frac{N\sum g_{obs}(t_{i})t_{i}-\sum g_{obs}(t_{i})\sum t_{i}}{N\sum t_{i}^{2}-{\left(\sum t_{i}\right)}^{2}} \end{array}$$ (2)

Thus, the observed rhythmic component of the signal can be obtained as follows:

$$r_{obs}(t_{i})=g_{obs}(t_{i})-a-bt_{i},\;i=1\ldots n$$ (3)

The rhythmic component parameters are obtained by minimizing the following Squared Residual Error (SRE):

$$SRE=\sum_{i=1}^{N}{\left[r_{obs}(t_{i})-A\;\text{sin}(2\pi Tt_{i}+\phi )\right]}^{2}$$ (4)

**Fig 1:**
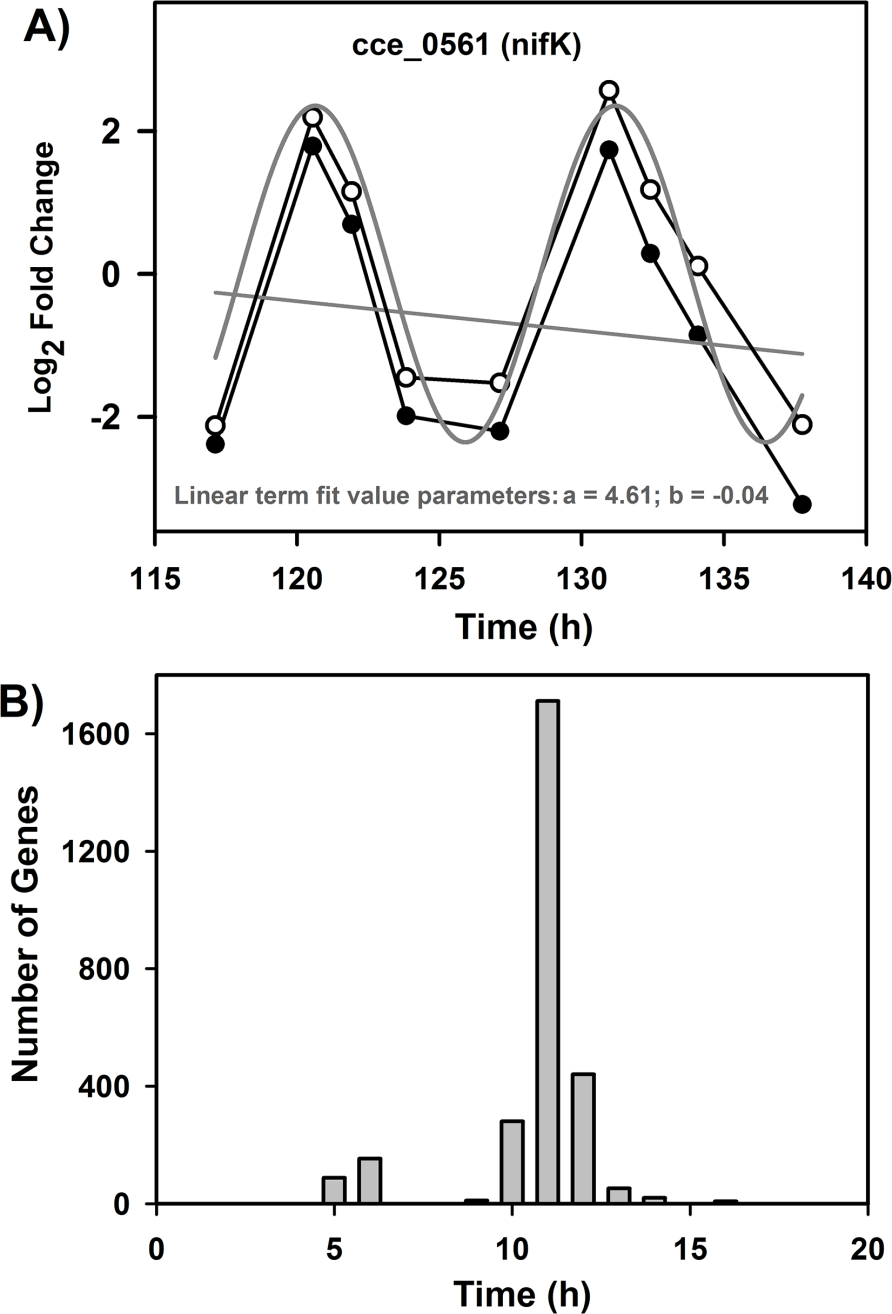
Sinusoidal model fitting to cyclic genes and estimation of model parameters. A: Results shown for *nifK* gene (cce_0561) as a representative under continuous light with the observed expression profile (●), linear term of the model (grey straight line, a + b*t from Eq (1)), data points after subtracting the linear term (○) and sinusoidal fit (grey sinusoidal curve). B: Histogram showing periodicity of oscillation for n = 2152 genes, deemed to be cyclic by sinusoidal model with a cut-off of 25 percent residual percent energy as a measure of goodness of fit.

Minimizing the SRE in (4) is a non-linear, non-convex optimization problem in the parameter set {*A*, *T*, *ϕ*}. Thus, local optimization procedures will need a good initial estimate to converge to a good solution. We solve this optimization problem as follows. First, we initialize the period T from 1h to 36h in step of 0.5h, while the other parameters are initialized as *A = max*_*i = 1*.*n*_ *r*_*obs*_ *(t*_*i*_ *)*, *ϕ = 0*. Matlab’s lsqcurvefit function is employed to minimize the error function from the initial guess. The period that results in the smallest residue is taken as the optimal period. An illustration for the model fitting process is given in Fig 1A. To assess the goodness-of-fit, we compute the residue percentage energy, defined as

$$\mathrm{RPE}=\frac{\sum_{i}{\left[r_{\mathrm{obs}}(t_{i})-A\sin(2\pi {Tt}_{i}+\phi )\right]}^{2}}{\sum_{i}{\left[A\sin(2\pi {Tt}_{i}+\phi )\right]}^{2}}$$ (5)

We deem RPE values of less than 25% to be a good fit.

### Fourier Score for Periodicity

When employing the sinusoidal model, there is always a possibility of missing those genes which have irregular patterns and whose oscillatory behaviour is significantly non-sinusoidal. To address this, we complement the sinusoidal model with a statistical test for periodicity, as detailed next. In order to test for statistical significance of periodicity in gene expression profiles, we employ the Fourier score, defined as:

$$F=\sqrt{{\left(\sum_{i=1}^{n}\text{sin}\;\omega t_{i}\cdot g_{obs}(t_{i})\right)}^{2}+{\left(\sum_{i=1}^{n}\text{cos}\;\omega t_{i}\cdot g_{obs}(t_{i})\right)}^{2}}$$ (6)

Where *ω* = 2*π* / *T*.

In a recent work [27], the Fourier score coupled with a permutation test was found to be effective for identifying gene behaving in a cyclic manner. Thus, here we also employ a permutation test with 10,000 permutations generated. The *p-*value for each gene is calculated based on the fraction of random profiles that has a Fourier score equal or higher than that of the gene being studied. It is to be noted that for this test, the period T must be provided a priori. We use the sinusoidal model to identify the periods. Since the number of tests being performed is large, we employ the false discovery rate (FDR) controlling procedure [28] to set a global threshold for *p-*value. In particular, the *p-*value of the genes are sorted in increasing order p_1_ *≤* p_2_ *≤ …*. Fix a false discovery rate q^*^, and identify k as the largest i for which *p*_*i*_ *≤* q^*^ i/m, where m is the number of tests, i.e. number of genes in this case. Then, all genes corresponding top_1_, p_2,…,_p_k_ are designated as periodic genes with period T.

## Results and Discussion

### Gene expression under constant light

Roughly 30% of *Cyanothece* genes oscillate under diurnal cycles [19]. Further, *Cyanothece* culture oscillates between photosynthesis and respiration with a cycle time of ~11 h under LL [17,18]. A select *Cyanothece* genes have also been shown to oscillate under LL [18]. Thus, it was of interest to determine if this was a genome-wide phenomenon and if the oscillations included cellular events other than metabolism. To that end, we obtained genome-wide gene expression time-course with DNA microarray during the photosynthetic, respiratory and transition phases of the culture under LL (S1 Fig). After a four-day entrainment, the culture takes ~20 h to settle into rhythmic oscillations. Thus, a total of 9 samples were collected from LL_21.1_ to LL_41.75_, covering two complete cycles of metabolic oscillations. Sample numbers 1, 5 and 9 are at the transition from photosynthetic to respiratory metabolism while samples 3 and 7 mark the reverse transition. Samples 2 and 6 are at the peak of respiration while samples 3 and 7 are at the peak of photosynthesis. Of the 5251genes, 2984genes showed > 2-fold change across the samples while 4755 genes satisfied a less stringent 1.5-fold change filter. When sorted by the average of raw intensity in the time course, the top 200 highly expressed ORFs comprised of components of ribosome, phosystems I and II, the nitrogenase complex, phycocyanin, allophycocyanin, cytochrome b6-f complex, ATP synthase, and hemeoxygenase (S1 Table). This list also contained over 100 hypothetical proteins suggesting that the unannotated genes may be involved in key cellular processes. Notably, majority of the top 200 highly expressed genes show > 1.5 fold change during the experiment. Highly expressed genes are also of interest for development of expression systems, promoters and ribosome binding sites for efficient heterologous protein expression.

### Detection of cycling genes and their periodicities

**Fig 2:**
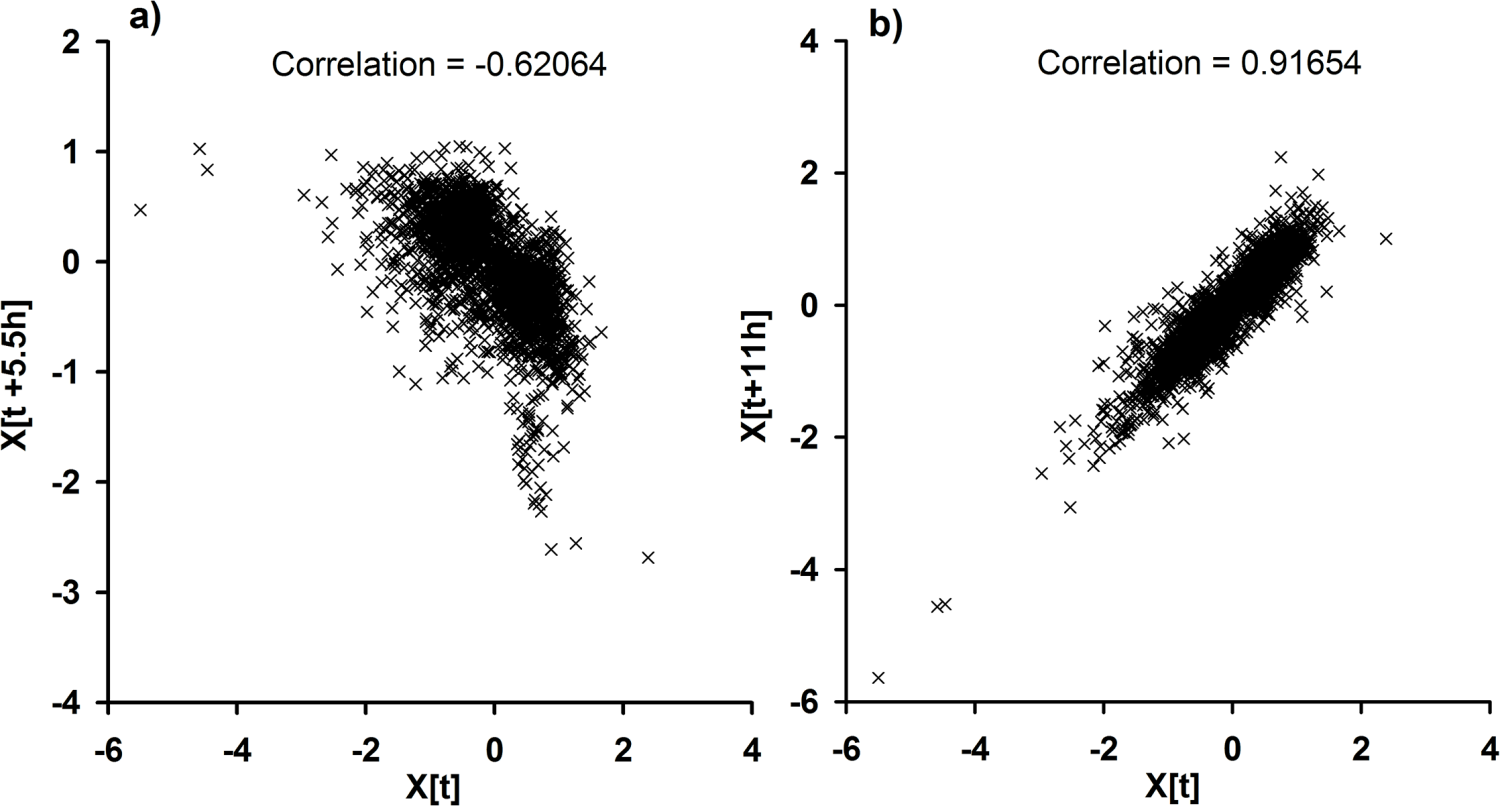
Scatter plot to demonstrate 11 h as the predominant periodicity among the cyclic genes. A scatter plot for 2152 genes that show satisfactory goodness of fit in the sinusoidal model with a randomly selected time point of t_121.92h_ as x-axis and its respective t+5.5h (t_127.13h_) or t+11h (t_132.42h_) as the y-axix. A: Scatter plot showing a negative correlation between the gene expression at t and t+ 5.5 h. B: Scatter plot showing a positive correlation between the gene expression at t and t+11 h.

We employ a two-stage procedure comprising a sinusoidal model [26,29] and Fourier score [27,30] to detect cycling genes and to estimate periodicity of cycling (*T*) as detailed in the materials and methods section. We first select 4755 of *Cyanothece* genes that satisfy 1.5-fold change threshold (Table 1) and delineate the linear trend or drift component from data for each gene (Fig 1A). A sinusoidal model is then fit and the residue percentage energy estimated as a measure of goodness of fit. A histogram of the estimated T values shows that majority of genes cycle with a T of ~ 11 h (Fig 1B). T value of 11± 1 his observed for 2710 genes of which 2152 genes show a satisfactory goodness of fit (Table 1). According to the Nyquist-Shannon sampling theorem [31], the minimum T value that can be accurately estimated with the current data is 5.6 h. Thus, the data supports the estimation of *T* of ~ 11h but not of 5 h as seen in the smaller peak in the histogram (Fig 1B). To further verify the main frequency of T ~ 11h, we provide the scatter plots for x(t) vs. x(t+11 h) and x(t) vs. x(t+5.5 h), where x(t)is the expression value of a gene at time t (Fig 2). There exists a strong positive correlation for x(t) vs. x(t+11 h) as expected, while the plot for x(t) vs. x(t+5.5 h) exhibits a strong negative correlation.

**Table 1:** Comparison of cyclic genes between *Cyanothece* 51142 and *Synechococcus* 7942.

	*Cyanothece* 51142		*Synechococcus* 7942
	Present Study	Data from [19]	Data from [32]
Light Regime^a^	LL	LD	LL
Number of genes	5251	4666	2662
Genes that pass the 1.5 fold filter	4755	1863	1218
Periodicity (T)	11±1	24±1	24±1
Model 1 predicted cyclic genes	2710	1024	636
Model 1 predicted cyclic genes satisfying T and RPE^b^ (25%)	2152	540	300
Model 2 predicted cyclic Genes	1705	1673	972
Genes that satisfy both Models	1202	540	300
Genes that satisfy either Models	2655	1673	972
Genes that satisfy none of the Models	1453	1133	672
Genes that satisfy Model 1 only	950	0	0
Genes that satisfy Model 2 only	503	1133	672

Two models, Sinusoidal model (Model 1) and Fourier score based model (Model 2) were used to test cyclic behavior of a given gene.

^a^LD: light dark cycles of 12 h each, LL: Continuous light.

^b^Residual Percentage Error

Having identified the major periodicity within the data being around 11 h, we next employ the statistical test based on Fourier score, as detailed in materials and methods section, to filter out those genes with significant periodicity in the vicinity of 11 h. In particular, for each gene, we perform 5 tests, with the period T fixed at 10 h, 10.5 h, 11 h, 11.5 h and 12 h, and record the smallest *p*-value. Next, the false discovery rate controlling procedure was carried out, with q*fixed at 5%. At this threshold, we identify 1705 genes as having a significant near 11 h periodicity. In total, using both procedures, we identify 1202genes satisfying both conditions. We deem this set of genes as having periodic expression profile for further analysis. A list of genes and gene expression data has been provided for genes that satisfy the 1.5-fold change threshold, the sinusoidal model, the statistical model or both models has been provided in S2 Table–S4 Table.

We performed analysis of cyclic genes with reported gene expression data for *Cyanothece* under diurnal cycles [19] and *Synechococcus* 7942 under constant light [32] (Table 1). Firstly, these studies report a relatively smaller fraction of genes that pass the 1.5-fold change threshold. We believe that this may be partially due to the differences in culturing conditions where we minimize light limitation by creating a simulated flashing light effect by growing the culture in turbulent regime [22,33]. In both the studies, the major periodicity element was of 24 h. Interestingly, only a small fraction of genes fit the sinusoidal model although a larger number of genes pass the Fourier score criteria.

### Equivalence between time of day in diurnal cycles and metabolic phase in continuous light

**Fig 3:**
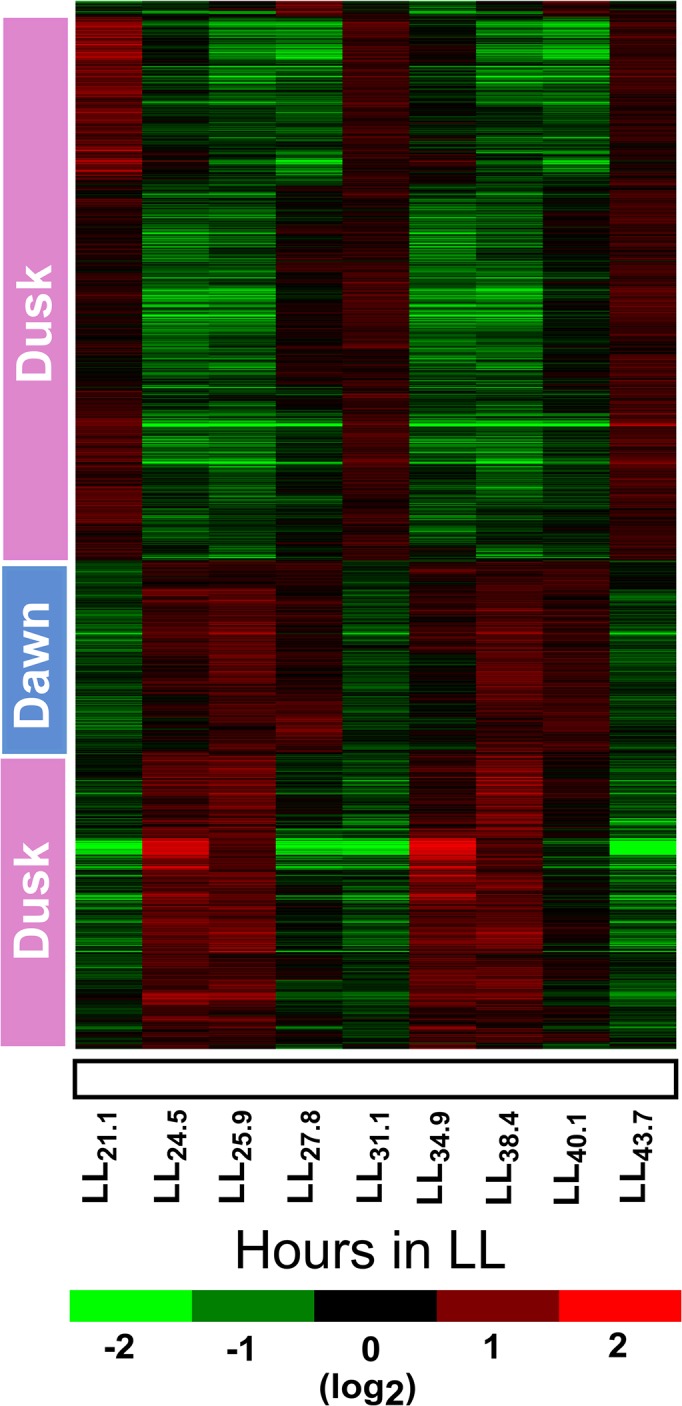
Heat map and hierarchical clustering of cyclic genes. Heat map was generated by performing hierarchical clustering for 1202 genes, which show 11h periodicity in oscillations and satisfy both Sinusoidal and Fourier Transform based models. Genes that peak under photosynthesis or respiration based metabolism are marked notionally as dawn peaking and dusk peaking, respectively. Normalized gene expression value, log_2_ (fold change) is plotted as per the scale bar shown below the x-axis.

**Fig 4:**
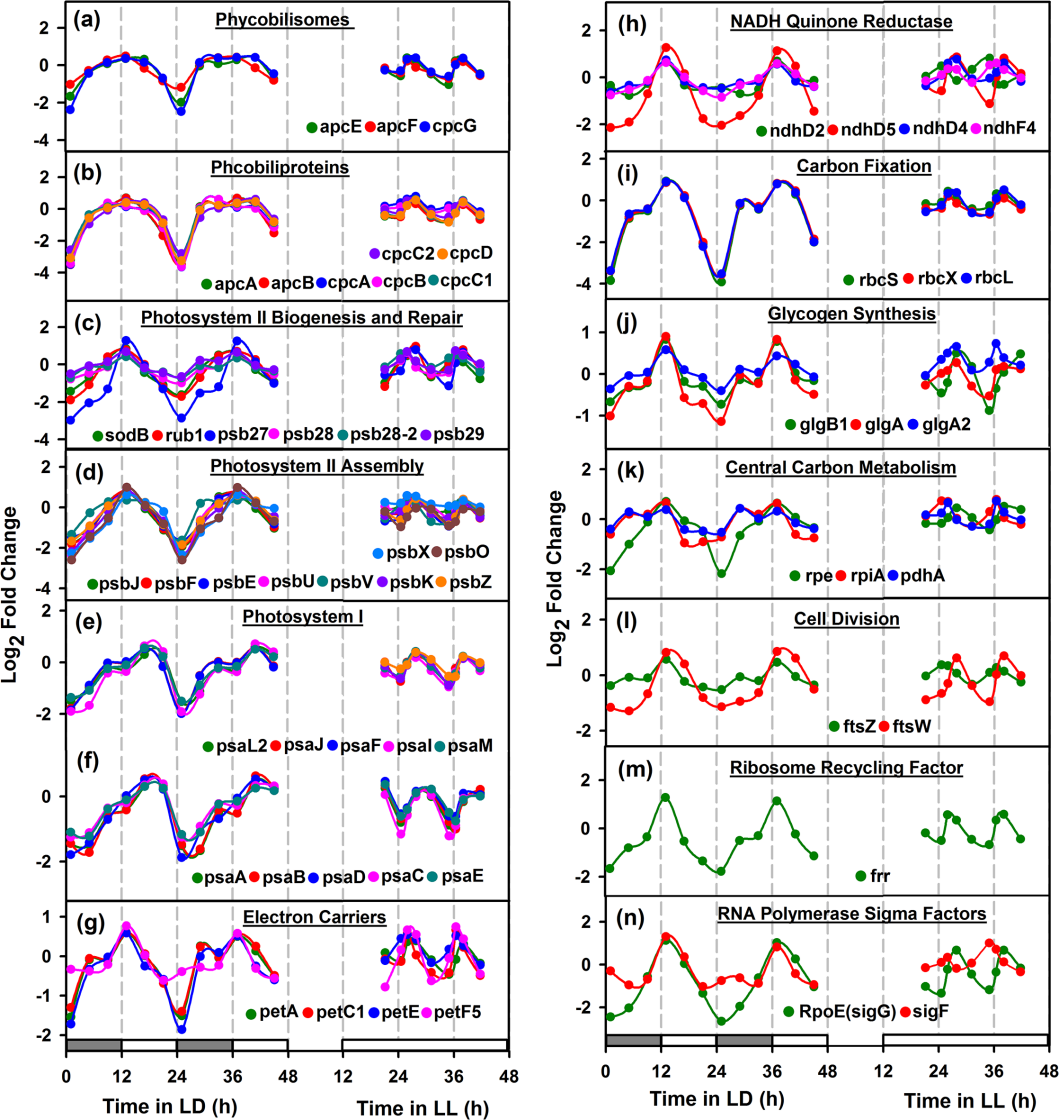
Day peaking oscillatory genes of *Cyanothece* 51142. Genes were selected based on 1.5-fold change cutoff and clustered using the gene expression data under continuous light (LL) (present study) and light/dark (LD) condition [19]. Panels (a) and (b) show the essential genes that are involved in the organization of Phycobilisomes and Phycobiliproteins; panels (c) and (d) show the genes that participate in biogenesis, repair and assembly of Photosystem II. Panels (e) and (f) refer to the genes that are involved in functioning of Photosystem I, peaking during the mid-day or mid of the photosynthetic phase. Panel (g)-(h) represent genes that participate in the electron transport chain and panels (i)-(k) represent the genes that play an important role in carbon fixation, glycogen synthesis and central carbon metabolism. Panels (l)-(n) show the genes that are involved in cell division, ribosome recycling, RNA polymerase sigma factor, where all up-regulate at dawn or during the onset or middle of the photosynthetic phase to support cell growth and maintenance. Grey and White filled and continuously white filled boxes in the X-axis, indicate the culture growth conditions of LD and LL, respectively.

**Fig 5:**
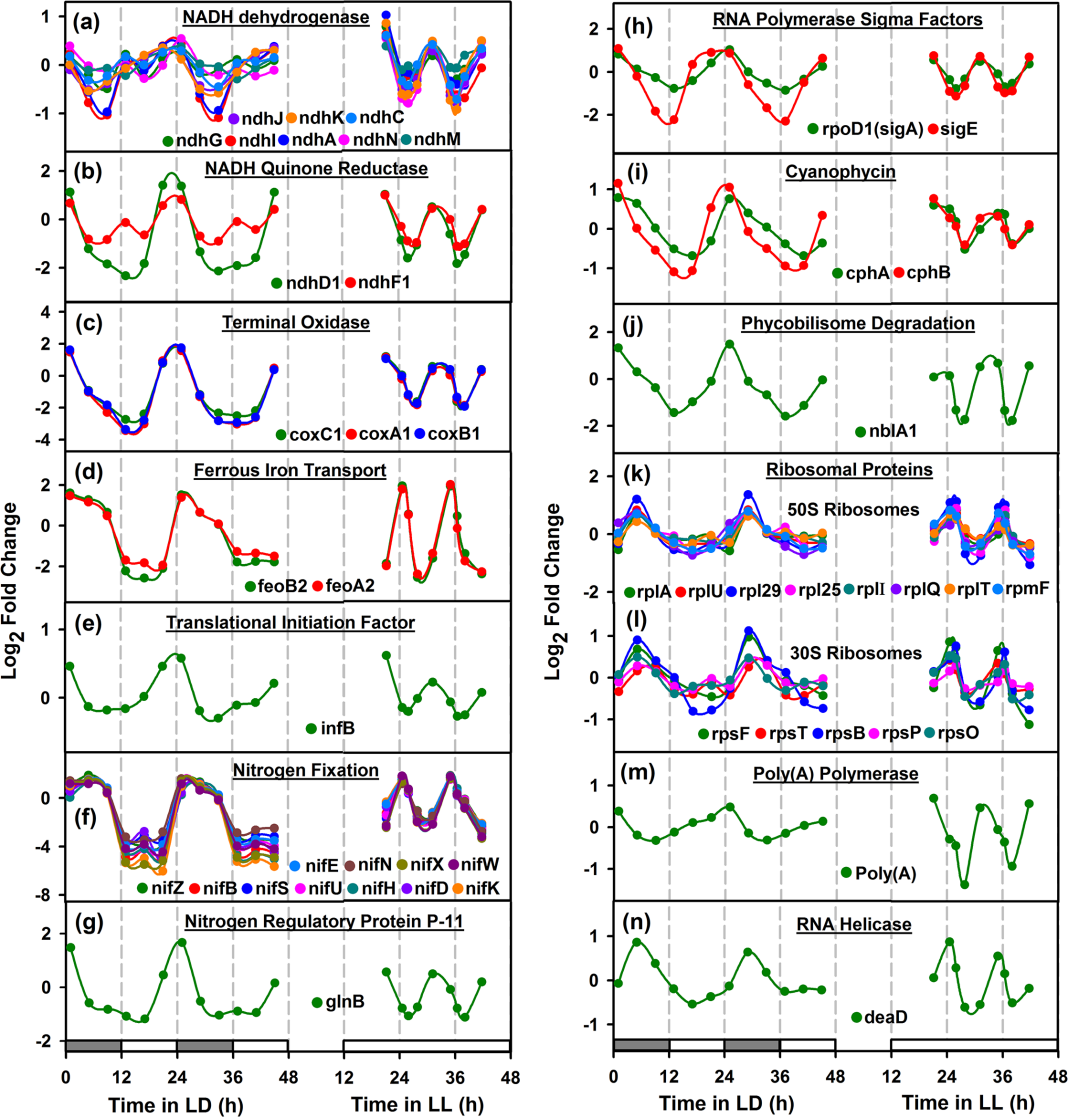
Night peaking oscillatory genes of *Cyanothece* 51142. Panels (a)-(c) show the genes that are involved in NADH dehydrogenase, NADH quinone reductase and terminal oxidase of oxidative phosophorylation pathway, peaking at dusk or prior to the onset of respiration phase. Panels (d)-(g) represent the genes that participate in the nitrogen fixation mechanism, such as ferrous iron transport, translational initiation, nitrogen fixing activity and nitrogen regulatory protein. Panels (h)-(j) show the genes that take part in RNA polymerase sigma factors, cyanophycin and phycobilisomes degradation. Panels (k)-(n) represent the genes that are involved in biogenesis of ribosomes and RNA metabolism, such as ribosomal proteins, poly(A) polymerase and RNA helicase. See legend to Fig 4 for other details.

**Fig 6:**
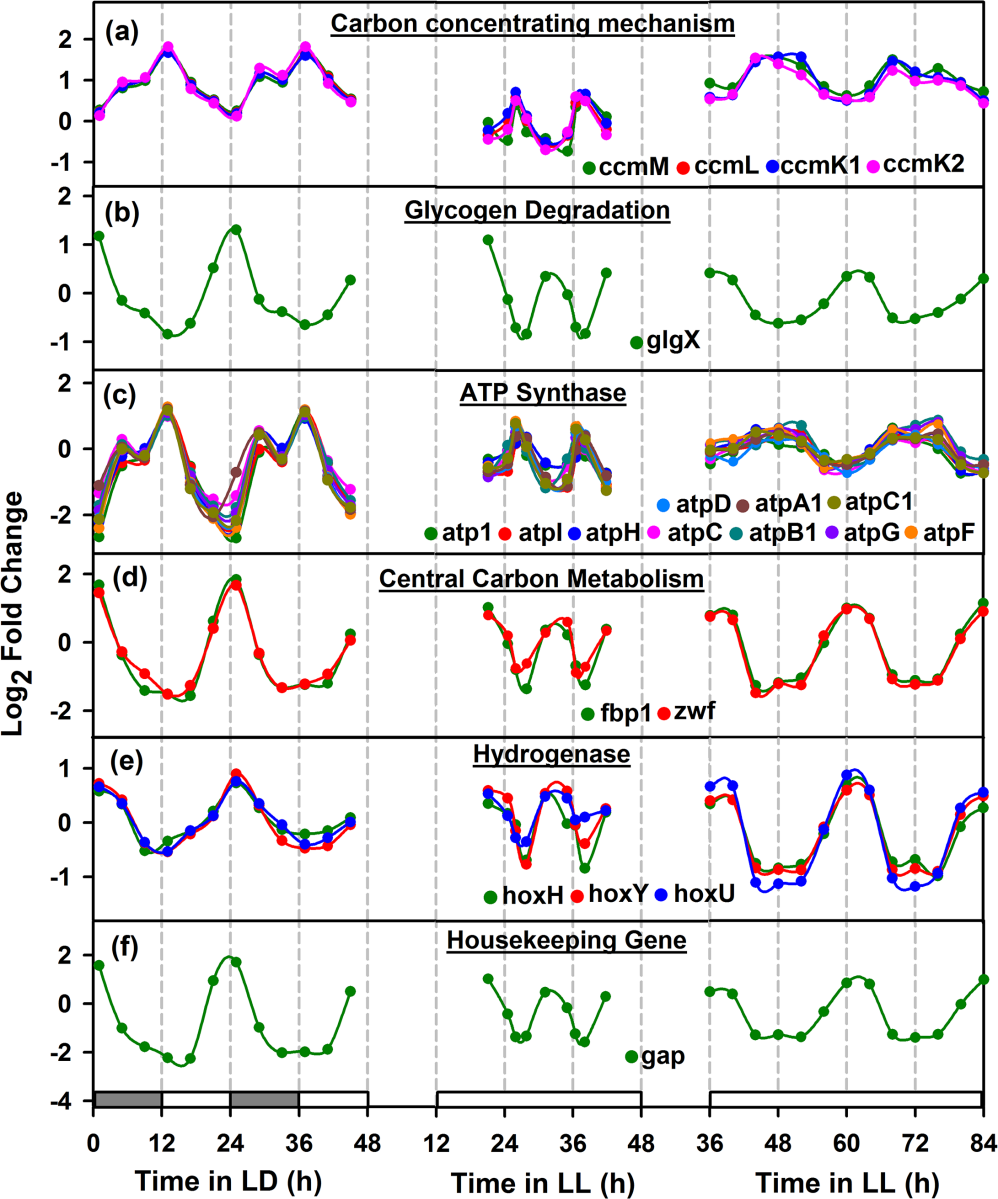
Comparison of oscillatory behavior of cyclic genes in nitrogen fixing (*Cyanothece* 51142) and non-nitrogen fixing organism (*Synechococcus* 8942). Cyclic genes were selected for *Cyanothece* 51142 and their homologs in non-nitrogen fixing *Synechococcus* 7942 (data from [32]). Panels (a) and (b) show the genes that are involved in day metabolism, such as carbon concentrating mechanism (CCM) and ATP synthase complex, peaking in the dawn or at the onset of photosynthetic phase. Panels (c)-(f) show the genes that peak during night metabolism, such as glycogen degradation, central carbon metabolism, hydrogenase and housekeeping gene, all peaking at dusk or at the onset of respiration phase or at subjective night. See legend to Fig 4 for details.

The net rates of CO_2_ uptake and O_2_ evolution (as estimated from exhaust gas analysis) allow us to monitor the metabolic phase of the culture in real time. Under diurnal cycles, the culture is observed in three broad categories of metabolic phases: (i) photosynthetic phase from dawn till ~1 h before dusk, (ii)respiratory phase from ~1 h pre-dusk to ~4 h into dark and (iii) maintenance phase with low respiration rates for the remaining ~8 h of dark cycle (S1 Fig). Under LL, both the photosynthetic and respiratory phases are shorter by 2–3 h each while the maintenance phase is absent thereby resulting in an 11 h cycle time. First, it was of interest to ascertain if the consecutive metabolic cycles under LL result in reproducible cycling of gene expression. A heatmap of the genes, upon hierarchical clustering and with correlation as a metric, shows peaking times for groups of genes (Fig 3). The genes that peak at a particular time during the first metabolic cycle also peak at an equivalent phase in the second cycle. Next, it was of interest to compare the peaking behaviour of genes under LL and LD and correlate this with the respective metabolic cycling. To that end, we selected genes that pass the 1.5-fold change threshold under diurnal cycles [19] and continuous light (this study) and subjected to K-means clustering with K = 40 clusters and correlation as a metric (S5 Table). Examination of representative clusters reveals that genes that peak at dawn also peak at the beginning of the photosynthetic phase under LL (i.e., data points 3 and 7) (e.g., Fig 4A–4I) while the mid-day peaking genes peak during late photosynthetic phase under LL (i.e., data points 4 and 8) (e.g., Fig 4E). By the same token, genes that peak at dusk peak at the beginning of respiration phase under LL (i.e. data points 1, 5 and 9) (e.g., Figs 5A–5C and 6B and 6D). Fig 5D and 5F are examples of genes that peak during early dark or mid-respiration under LL (points 2 and 6). This clearly establishes the metabolic and gene expression equivalence between the various phases of the 11 h cycle under LL with the time of day under LD. We then use the Biological Networks Gene Ontology tool (BiNGO) to systematically check for overrepresentation of Gene Ontology (GO) terms in the 40 clusters [34]. We find that a number of clusters are enriched in Functional category or GO terms (Table 2). Some of the enriched functions included the expected categories of photosystem I, nitrogen fixation and proton-transporting ATP synthase complex.

**Table 2:** Assessment of overrepresentation of Gene Ontology terms among the gene clusters of *Cyanothece* 51142^a^.

Cluster	Number of genes	Overrepresented Gene Ontology term^b^	Corrected p-value
5	55	Photosynthesis	2.70E-35
6	80	NAD(P) transhydrogenase activity	9.67E-04
7	54	Regulation of cell morphogenesis	2.97E-03
15	9	Inorganic phosphate transmembrane transporter activity	7.19E-08
23	78	Photosystem I	9.08E-09
24	80	Nitrogen fixation	8.64E-23
27	30	Electron transport chain	1.19E-03
28	62	rRNA binding	5.41E-34
29	53	Acetyl-CoA carboxylase complex	6.98E-04
30	60	Copper-transporting ATPase activity	3.53E-05
31	68	Plasma membrane proton-transporting ATP synthase complex	7.04E-13
37	64	NADH dehydrogenase (ubiquinone) activity	1.13E-08

^a^Genes were clustered using the K-means algorithms. Genes of *Cyanothece* 51142 that satisfy the 1.5-fold change filter in the gene expression data under LL (9 time points) (present study) and LD (12 time points) (data from [19]) were subjected to K-means clustering using K = 40 clusters.

^b^Overrepresentation of Gene Ontology terms was performed using Biological Networks Gene Ontology tool (BiNGO) [34].

### Oscillation between carbon and nitrogen uptake

*Cyanothece* 51142 oscillates between carbon uptake during light phase and nitrogen uptake during dark phase under diurnal cycles [4,17,35]. This arrangement suits the organism as the two processes are incompatible. We and others have reported oscillation between photosynthesis and respiration for this strain even under continuous light (S1 Fig). Photosynthesis is accompanied by upregulation of genes associated with photosynthesis, carbon uptake and glycogen synthesis (Fig 4). On the contrary, the respiration phase is characterized by upregulation of genes involved in glycogen degradation, TCA cycle, oxidative pentose phosphate pathway and nitrogen fixation (Fig 5). We believe that these oscillations serve the purpose of maintaining a healthy Carbon-Nitrogen (C/N) ratio within the cells under continuous light. Since the nitrogen fixation and photosynthesis (or carbon fixation) processes are not compatible, lack of oscillations may lead to continuous carbon fixation and nitrogen starvation or vice versa.

### Genes peaking during photosynthetic phase

Fig 4 shows peaking behavior of representative genes that peak during the photosynthetic phase under LL. Photosystems (PS) I and II, accessory proteins and pigments, RuBisCO, among others fall in this category. Phycobilisomes or the light harvesting antennae of cyanobacteria are comprised of phycocyanin, allophycocyanin and other accessory proteins. Components of phycobilisomes (Fig 4A and 4B), PS II (Fig 4C and 4D), RuBisCO subunits (Fig 4I) get upregulated during early photosynthetic phase (time points 3 and 7) under LL. Components of PS I, on the other hand, exhibit delayed response and peak during mid-day under LD and mid-photosynthetic phase (time points 4 and 8) under LL (Fig 4E and 4F). Fig 4 also includes examples of genes that are dawn peaking under LD but peak at mid-photosynthetic phase under LL (Fig 4J and 4N). For instance, the oscillations in glycogen synthesis genes (Fig 4J) show the aforementioned pattern of oscillations. The expression of glycogen synthesis genes is possibly dependent on the intracellular glycogen concentration, which also oscillates at ~11h frequency under LL, however with reduced amplitudes[18]. So, possibly in absence of external constraints of light/dark cycles, the glycogen synthesis genes expression is driven solely by the depletion of intracellular glycogen content.

### Carbon concentrating mechanism

**Fig 7:**
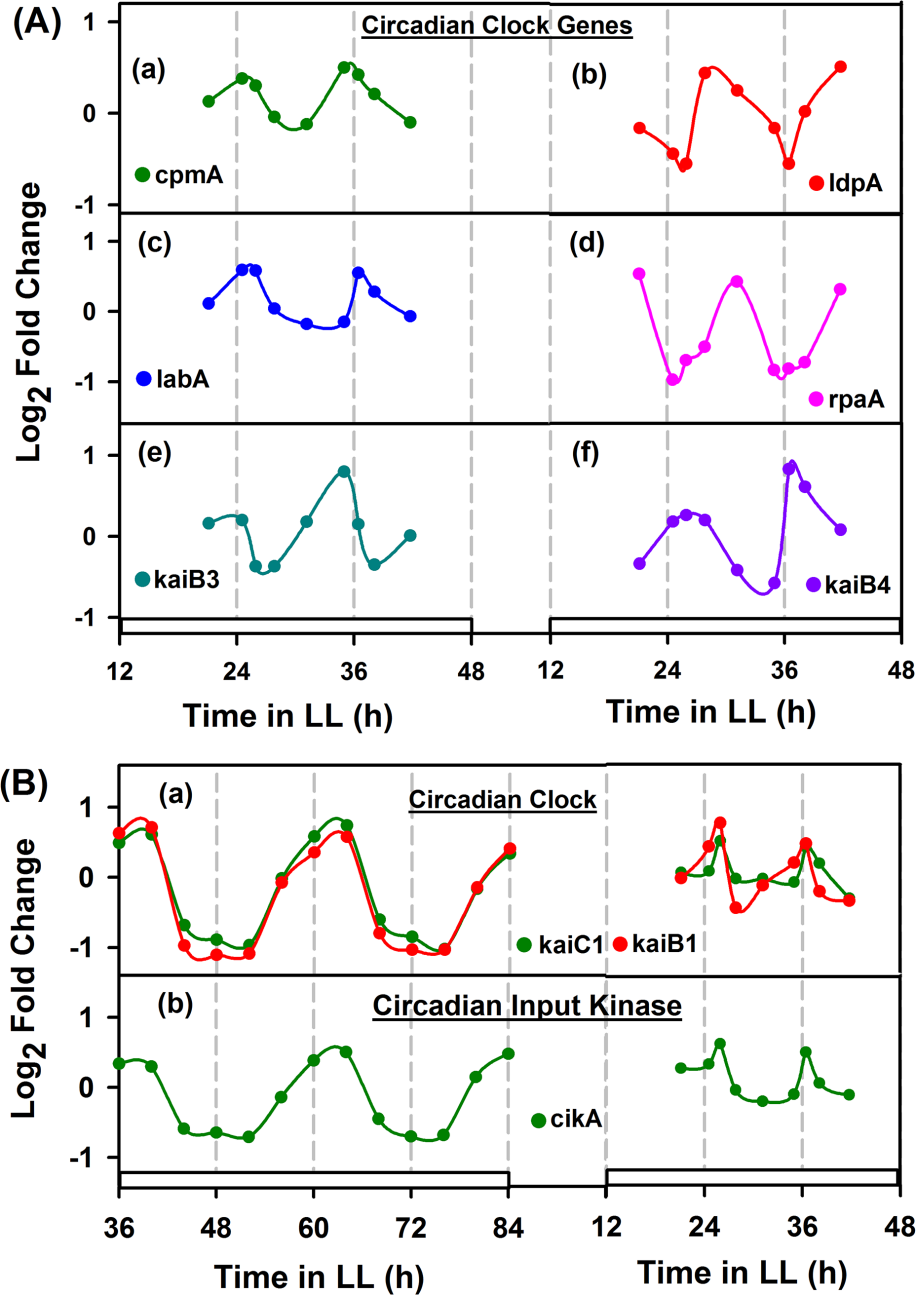
Oscillation in circadian clock genes. Selection of clock genes in *Cyanothece* 51142 was performed by considering the homologues of *Synechococcus* 7942. A: Panel (a-f) shows the oscillation of clock genes in *Cyanothece*51142 under continuous light (LL), involving in input pathway (l*dpA*), output pathway (c*pmA*, l*abA*), master clock regulator (r*paA*) and core structural clock mechanism (k*aiB3* and k*aiB4*). B: Panel (a) and (b) shows the comparison of clock genes involved in core structural clock mechanism (k*aiC1* and k*aiB1*) and input pathway of clock (c*ikA*), showing 24h periodicity for *Synechococcus* 7942 and approximately 11 h periodicity for *Cyanothece* 51142 under continuous light. White filled boxes in the X-axis, indicate the culture growth under LL condition.

Many cyanobacteria have devised a carbon concentrating mechanism (CCM) through which the process of carbon fixation occurs within the proteinecious micro-compartments called carboxysomes. Carboxysomes encapsulate the principle caboxylating enzyme ribulsose bis-phospahte carboxylase/oxygenase (RuBisCO) for efficient enzymatic activity of this enzyme mediated by elevated CO_2_ concentration around it. The gene expression pattern of the CCM genes and that of the RuBisCO precedes the beginning of the photosynthesis phase as well as the expression of the photosystem genes (Figs 4I and 7A). This should be for facilitating the assembly of the carboxysome and the encapsulation of RubisCO to ensure that the carbon fixation machinery is ready when the photosynthesis begins. Interestingly, in *Cyanothece* 51142, the size of carboxysomes is predicted to be around 250–600 nm [36], which is significantly larger than those in other cyanobacteria which ranges around 100 to 200 nm [37,38]. Intriguingly, the expression patterns of many CCM genes such as *ccmM*, *ccmL*, *ccmK1* and *ccmK2* coincide with the expression of *nif* genes, suggesting the possibility of the micro-compartmentalisation of nitrogenase as a mechanism for its protection from oxygenic inactivation. Co-localization studies of nitrogenase with the carboxysome shell proteins in *Cyanothece* 51142 should yield a better explanation on oxygen protection mechanism in this organism, especially during growth under constant light.

### Housekeeping Genes

Housekeeping genes are genes involved in basic cellular functions and are understood to be constitutively expressed. However several genes conventionally believed as housekeeping have been observed to show an oscillating pattern of expression in cyanobacteria [8] and it is hence deemed appropriate to not go with a priori determination of constitutive genes while analysing the transcriptome datasets in cyanobacteria [39]. Under the present study we observe an oscillating gene expression of *gap* (Fig 6F), a gene annotated as housekeeping, similar to the peaking of sigma factors. Other designated housekeeping genes (S2 Fig) show an expression pattern overlapping with *nif* genes under LL. Interestingly, these genes peak around the middle of the dark phase under LD cycles, unlike the *nif* gene cluster which peak at the onset of the dark phase under LD[19]. This could be indicative that under ultradian rhythm, the resolution of the gene expression pattern might be less resolved as compared to that in the circadian rhythm under LD.

### Dusk Peaking Genes

To analyze genes that peak during dusk/respiration phase, we plotted the representative genes that show oscillatory behaviour under LD and LL conditions (Fig 5). Interestingly, we noted that the genes such as NADH dehydrogenase; NADH quinone reductase; terminal oxidase; RNA polymerase sigma factor; translational initiation factor (*infB*) and nitrogen regulatory protein P-11 (*glnB*), showed peaking during early respiration (time points 1 and 5) (Fig (5A–5C), 5(E), 5(G) and 5(H)). However, ferrous iron transport and nitrogen fixing genes showed peaking at mid or late respiration (time points 2 and 6) (Fig 5(D) and 5(F)). Furthermore, the cyanophycin genes peaked concomitantly with the nitrogenase activity (Fig 5(I)), thereby serving as a key nitrogen reserve for the organism [40]. We also noticed that the phycobilisome degradation gene (*nblA1*), up-regulating during respiration phase (time points 2 and 6) (Fig 5(J)), to control photosynthetic activity during nitrogen fixation [41,42].

### Ribosome biogenesis at midnight

#### RNA processing (DEAD box RNA helicase)

DEAD box RNA helicases are widespread in all kingdoms of life and are known to be cold-induced in some prokaryotes. They are implicated in varied functions including rearrangement of RNA secondary structure that are stabilized especially at low temperatures, Ribosome biogenesis [43], and RNA turnover [44]. Deletion of the gene in *Synechocystis* shows deleterious effects at all temperatures but more prominent effects are seen at low temperatures[45]. In *Synechocystis*, the *crhR* gene has been further shown to be is regulated by the redox status of the electron transport chain. The electron carrier redox state was initiated by either photosynthetic light harvesting or glucose metabolism. Interestingly, *Cyanothece* contains cce_4436 (*deaD*), a close homolog of *crhR*, which oscillates in LD as well as in LL. Under both conditions, the *deaD* gene peaks concomitantly with the ribosomal proteins (Fig 5N). This peak occurs at midnight under LD or during respiratory burst under LL. This first of all suggests that the gene is not merely light responsive but may be responsive to the metabolic phase or redox state. Secondly, the correlation with ribosomal proteins strongly suggests a role of *deaD* gene in ribosome biogenesis.

#### Light / Phase sensitive Sigma Factors / lrtA

It has been shown that some of the sigma factors are light activated while some others are light repressed in *Synechocystis* sp. PCC 6803[46],[47]. For example, SigE is light induced and dark repressed while SigD is light induced. SigB is light repressed and dark induced. Other sigma factors were not found to be responsive to light. In *Cyanothece*, we find a number of sigma factors to oscillate under LD as well as LL conditions (Figs 4 and 5). We observe that *sigD* and *sigE* to be upregulated by the end of the light phase in LD or toward the end of photosynthetic phase in LL. Likewise, *sigA* and *sigH* peak at dusk *sigC*, *sigF*, *sigG*, *sigJ* peak at dawn while *sigB* peaks at midday. In *Synechocystis* sp. PCC 6803, *sigB* is known to be heat shock inducible [47]. However, in *Cyanothece*, we find *sig B* to oscillate both in LD and LL with peaking at mid-day or during photosynthetic phase. This first of all suggests that the sigma factors are not merely responding to light (or dark) or heat shock but also to changes in the metabolic phase of the culture. Interestingly the peaks in expression of the clock genes is followed by peaks for sigma factors such as *sigA*, *sigB*, *sigE* and *sigJ*, indicating that these sigma factors may be taking input from the clock proteins and reiterating role of circadian clock in global gene expression regulation in cyanobacteria. Although our sampling frequency does not allow us to identify precise phase behaviour of the various sigma factors, the results suggest that the sigma factors respond to the metabolic phase of the culture and may be either controlled by the energy, carbon and nitrogen status of the cell directly or through intermediaries such as the circadian clock. The light repressed protein *lrtA* has been shown to be down-regulated in response to white light in *Synechocystis* 6803 [47] and *Synechococcus* 7002 [48]. However, we find that the *lrtA* transcript oscillates in LD and LL and gets upregulated in light or under photosynthetic phase and peaks at dusk.

#### Programmed RNA degradation at dusk

RNA degradation used to be considered as a nuisance in laboratory protocols until two decades ago. It is now emerging that RNA degradation is programmed and is catalyzed by elaborate machinery involving a degradosome. We find a number of genes involved in RNA degradation to peak at dusk. For example, the poly(A) polymerase involved in RNA degradation peaks at dusk or relative dusk under LL (Fig 6M). This raises a question whether this suggests expedited RNA degradation at dusk, possibly for a midnight ribosome biogenesis or to shut off all the daytime enzymes. Further, we also notice that the genes that peak at dusk or early dark do see a short half-life compared to those that peak at mid-night or at dawn.

### Comparison of *Cyanothece* 51142 diurnal metabolism with *Synechococcus* 7942

To demonstrate the diurnal metabolism under continuous light, we analyzed the transcriptomic data for the present study with *Cyanothece* 51142 [19]and *Synechococcus* 7942 [32] under LD and LL conditions, respectively. We considered only the genes that show oscillation, such as from carbon concentrating mechanism, glycogen degradation, ATP synthase complex, central carbon metabolism, hydrogenase and housekeeping gene (Fig 6). Interestingly, we noticed *Cyanothece* 51142 and *Synechococcus* 7942 under respective LD and LL conditions showed ~24h periodicity, while the present study (LL) reported ~11h periodicity. Genes involved in carbon concentrating mechanism (CCM) (i.e., *ccmM*, *ccmL*, *ccmK1* and *ccmK2*) and the core genes of ATP synthesis complex (i.e., *atp1*, *atpI*, *atpH*, *atpC*, *atpB1*, *atpG*, *atpF*, *atpD*, *atpA1* and *atpC1*), showed peaking at dawn or subjective dawn (Fig 6(A) and 6(B)).

On the contrary, at night metabolism, the genes from glycogen degradation (*glgX*), central carbon metabolism (*fbp1* and *zwf*) and hydrogenase complex (*hoxH*, *hoxY* and *hoxU*) (Fig 6(B) and 6D–6E), reported peaking at early dusk or subjective dusk. Of these, *glgX* indicated the carbohydrate degradation activity to assist nitrogen fixation; genes *fbp1* and *zwf* represented the induced activity of oxidative pentose phosphate cycle under dark anoxic condition and hydrogenase complex genes suggested the characteristic reduction of protons to produce hydrogen under dark fermentative condition. Furthermore, we analyzed the housekeeping gene glyceraldehyde 3-phosphate dehydrogenase (*gap*), an internal control gene which is commonly used in the experiment [49], showed a strong expression at dusk (Fig 6(F)) suggesting that organism undergoes a controlled temporal separation of day/night cell activities in continuous light.

### The clock genes and the input and output pathways

In cyanobacterial species, *Synechococcus* 7942 was the first model organism reported to have an established circadian clock mechanism. To elucidate the circadian clock behavior in *Cyanothece*51142 under LL condition, we analyzed the transcriptomic data of central core oscillator genes (*kaiA*, k*aiB* and *kaiC*) and homologues of *Synechococcus*7942 (*cikA*, *cpmA*, *ldpA*, *labA* and *rpaA*) that are majorly involved in input/output pathway of circadian clock system (Fig 7A and 7B). We considered only the genes that show 1.5-fold change in the expression level for the analysis.

In *Synechococcus* 7942, it has been reported that most of the genes show circadian expression at subjective dusk under continuous light [8]. However, in case of *Cyanothece* 51142, we had observed that the corresponding *kai* genes homologues oscillate in an inverse pattern to that in *Synechococcus*7942 [18]. In the present study, we noticed similar type of induced expression for *kai* genes (*kaiC1*, *kaiB1* and *kaiB4*) during the day for *Cyanothece* 51142 (7B(a) and 7A(f)) and up-regulation in the subjective night for the genes *kaiC1* and *kaiB1* in *Synechococcus*7942 (Fig 7B(a)) under LL condition. Interestingly, we also observed *kaiC1*plays a dominant role in the circadian clock in *Cyanothece* 51142 under our experimental conditions, as *kaiC2*does not exhibit similar oscillations (data not shown) under the culturing conditions used for the present study.

On analyzing the homolog c*ikA* (cce_4751), an essential input pathway component for cell division, circadian phase resetting and circadian period regulation [50], showed up-regulation in the early dawn for *Cyanothece*51142 and at subjective dusk in *Synechococcus*7942 (Fig 7B(b)). Another *Synechococcus* homolog *ldpA* (cce_2350), a ‘light-dependent period A’ input clock gene, reported high expression in the late photosynthetic phase (Fig 7A(b)), suggested that it may act as ‘periodosome’ in adjusting the circadian period length according to the light intensity. It has been also reported that the *ldpA* gene in *Synechococcus*7942, functions as ‘periodosome’ by modulating the circadian periodicity of length, either as ‘short period’ at high light or ‘long period’ under low light conditions [51].

We also noticed that the sensory histidine kinase gene (s*asA*), a positive input pathway regulator of circadian gene expression showed arrhythmic oscillation (data not shown), possibly due to some artifacts during sample processing or transcriptomic analysis or combined thereof. However, it has been shown that *sasA* oscillates along with its cognate response regulator *rpaA* under LL condition [18], suggesting that the possibility of KaiC-SasA-RpaA positive interaction pathway in mediating the output pathway of circadian clock. In supportive of this, the master clock regulator r*paA* showed up-regulation in the late photosynthesis phase (7A(d)). We have also suggested a possible role of SasA in signaling the onset of nitrogenase gene complex expression from the synchronized expression of *nif* genes and *sasA* under LD as well as LL [18].

Interestingly, for the homolog *labA* (cce_3317), a negative regulator of circadian gene expression, we find up-regulation in the subjective night (7A(c)). It has been also reported that *labA* in *Synechococcus* 7942, peaks in the subjective night to regulate the auto-phosphorylation of *kaiC* through negative feedback mechanism [52]. While analyzing the homolog c*pmA* (cce_2642), an output pathway component of circadian clock, showed high expression at dusk (Fig 7A(a)), which suggested that it might play in regulating the phase or amplitude of the output rhythm.

## Conclusions

**Fig 8:**
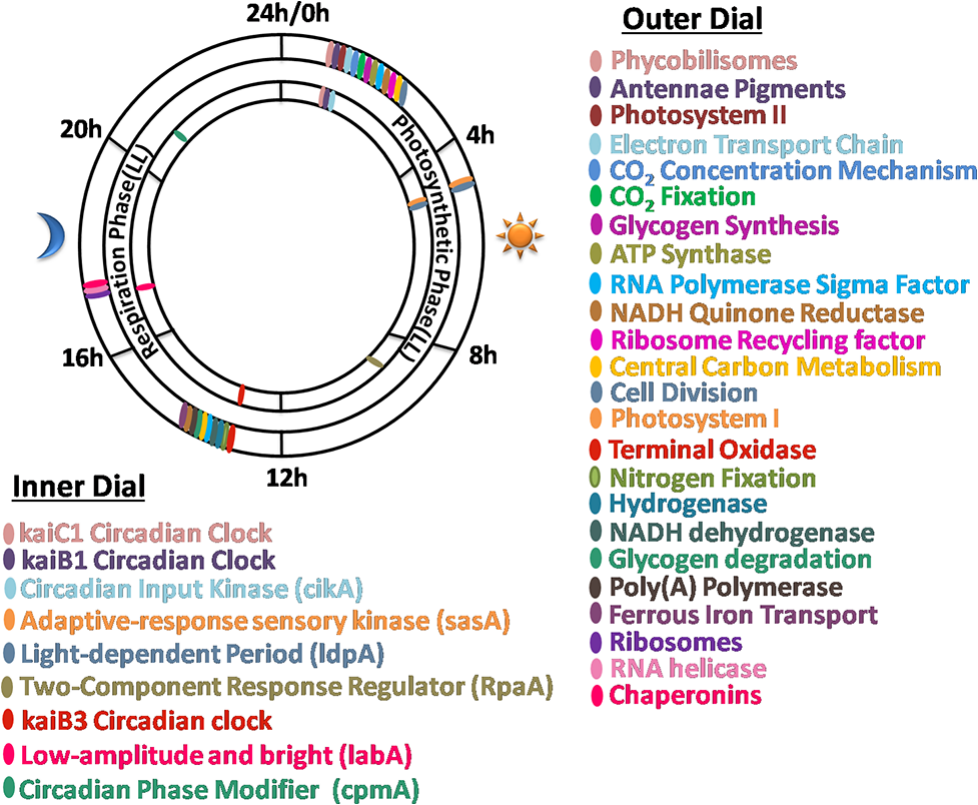
Peaking behavior of *Cyanothece* 51142 genes under diurnal cycles (LD) and continuous light (LL). Clock dial shows the cellular events that peak at a particular metabolic phase under LL or at a particular time of the day under LD. Cellular events or broad cellular functions are marked on the dial if majority of genes associated with it peak at the same time or at the same phase, under LD and LL, respectively.

A significant fraction of genes of *Cyanothece* 51142 oscillate under diurnal cycles as well as continuous light. Fig 8 summarizes the peaking behavior of a representative set of genes. Given the sampling frequency, the time resolution of this analysis is 4 h under LD and 2 h under LL. A large fraction of genes peak either in the first 4 h of light or the first 4 h of dark. A few genes peak in the middle of the day or night and hardly any of the metabolic genes peak late in the day or night. We find a strong relationship between the time of the day when genes peak under LD and the metabolic phase under which genes peak under LL. Based on the exhaust gas CO_2_ and O_2_ analysis, we broadly define the metabolic phases as photosynthetic phase, respiratory phase and the transition phases. The genes that peak at dawn and dusk under LD also peak at the beginning of photosynthesis and respiratory phases, respectively under LL. The ultradian rhythm in global gene expression further confirms it as the free running period for this organism in contrast to the hypothesis of such rhythm arising as an exception under specific growth conditions such as under elevated CO_2_ and resulting independent of its circadian clock [17]. The present study hence puts this critical phenomenon in perspective for further understanding and applying the core regulatory mechanism(s) in this model organism towards engineering it for channelizing photosynthetic energy towards nitrogen fixation and hydrogen production.

## Supporting Information

S1 Fig Metabolic oscillations in *Cyanothece* 51142 organism. Figure shows the online measured exit gas O_2_ profile for *Cyanothece* 51142, indicating 24h and 11h periodic oscillations under light-dark (LD) and continuous light(LL), respectively. Open circles with linear texts in the online profile, denotes the time points at which samples were collected from two diurnal cycles for gene expression analysis under LL condition. Boxes in white and grey filled and continuously white filled in the X-axis, indicate the culture growth conditions of LD and LL, respectively. A representative profile was considered for plotting from three replicates of the experiment.
(TIF)

S2 Fig Oscillation in housekeeping genes. A set of housekeeping genes that reported in *Lyngbyamajuscula*[53], which show cyclic oscillationsfrom the transcriptomicdata of light/dark (LD) condition[19] and present study (LL) were selected for the analysis. Panel (a-d) shows the oscillation of housekeeping genes, involved in carbohydrate transport, chaperonins and ribosomal proteins. Grey and White filled and continuously white filled boxes in the X-axis, indicate the culture growth conditions of LD and LL, respectively.
(TIF)

S1 Table Dataset of raw average intensity values. Average for the raw intensity values across nine samples was calculated for the present studyunder continuous light (LL). Sorting of the average raw intensity values was performed at higher order to list the highly expressed ORFs associated to various cellular functions.
(XLS)

S2 Table Dataset of cyclic genes for *Cyanothece* 51142 under continuous light. Transcriptomic data from the present study (LL condition) was considered, to identify the cyclic genes that show a periodicity (T) of ±11h, using Sinusoidal and Fourier Transform parameter fitting model approach. A Residual Percent Energy (RPE) of 25 percent was used as aconstraint in the model to select only the genes that show goodness of fit. To analyze the prediction results, in both Model1 (Sinusoidal) and Model2 (Fourier Transform), the genes that satisfy both the models, either of the models, none of the models, only Model1 and only Model2 were identified and listed with the corresponding gene expression values and gene annotation details.
(XLS)

S3 Table Dataset of cyclic genes for *Cyanothece* 51142 under Light/Dark cycle. Transcriptomic data from light/dark (LD)conditionwas considered, to identify the cyclic genes that show a periodicity (T) of ±24h, using Sinusoidal and Fourier Transform parameter fitting model approach. The genes that satisfy both the models, either of the models, none of the models, only Model1 and only Model2, were identified as similar to that of mentioned therein in S2 Table.
(XLS)

S4 Table Dataset of cyclic genes for *Synechococcus*7942 under continuous light. Transcriptomic data from continuous light condition (LL) [32] was considered, to identify the cyclic genes that show a periodicity (T) of ±24h, using Sinusoidal and Fourier Transform parameter fitting model approach. For identification of genes that satisfy both the models, either of the models, none of the models, only Model1 and only Model2, similar methodology was adopted as mentioned therein in S2 Table.
(XLS)

S5 Table Gene clusters of time-course profile expressed genes. Clusters of K = 40 were generated for *Cyanothece* 51142 by K-means method using 1.5 fold gene expression data from light/dark (LD) condition [19]and the present study (LL), which then mapped with the homologous genes from continuous light (LL) data for *Synechococcus* 7942[32]. Homology for *Synechococcus* 7942 was performed using BLAST with an e-value cut-off of 1E-5 against the protein sequences of *Cyanothece* 51142. BLAST score density values were additionally included as a standard measure to evaluate the correctness of identified homologues. To highlight the operonic genes in the clustered data, the operon information for *Cyanothece* 51142 from Cyano Operon database [54]was included for each group of theidentified K = 40 clusters.
(XLS)

S6 Table Gene clusters of time-course profile expressed genes. Clusters of K = 30 were generated for *Cyanothece* 51142 by K-means method using 1.5 fold gene expression data from light/dark (LD) condition [19]and the present study (LL), which then mapped with the homologous genes from continuous light (LL) data for *Synechococcus* 7942[32]. Homology for *Synechococcus* 7942 was performed using BLAST with an e-value cut-off of 1E-5 against the protein sequences of *Cyanothece* 51142. BLAST score density values were additionally included as a standard measure to evaluate the correctness of identified homologues. To highlight the operonic genes in the clustered data, the operon information for *Cyanothece* 51142 from Cyano Operon database [54]was included for each group of the identified K = 30 clusters.
(XLS)
