# Supplementary figures and images for: Coupling of Cellular Processes and Their Coordinated Oscillations under Continuous Light in Cyanothece sp. ATCC 51142, a Diazotrophic Unicellular Cyanobacterium
Source: PLoS One. 2015 May 14;10(5):e0125148. doi: 10.1371/journal.pone.0125148 (PMC4431719; doi:10.1371/journal.pone.0125148)

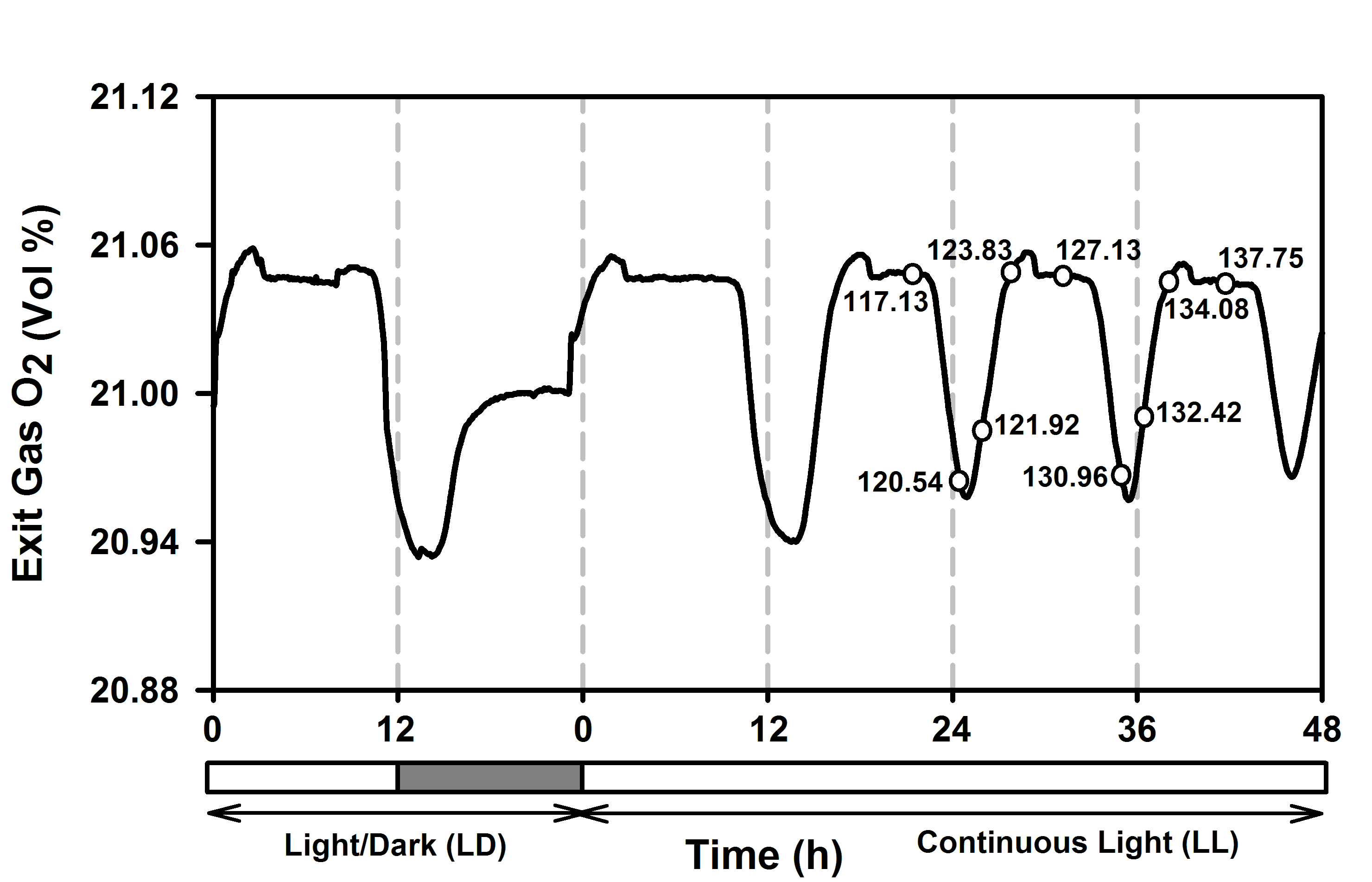

Supplement: S1 Fig — Figure shows the online measured exit gas O2 profile for Cyanothece 51142, indicating 24h and 11h periodic oscillations under light-dark (LD) and continuous light(LL), respectively. Open circles with linear texts in the online profile, denotes the time points at which samples were collected from two diurnal cycles for gene expression analysis under LL condition. Boxes in white and grey filled and continuously white filled in the X-axis, indicate the culture growth conditions of LD and LL, respectively. A representative profile was considered for plotting from three replicates of the experiment. (TIF) [file pone.0125148.s001.tif]

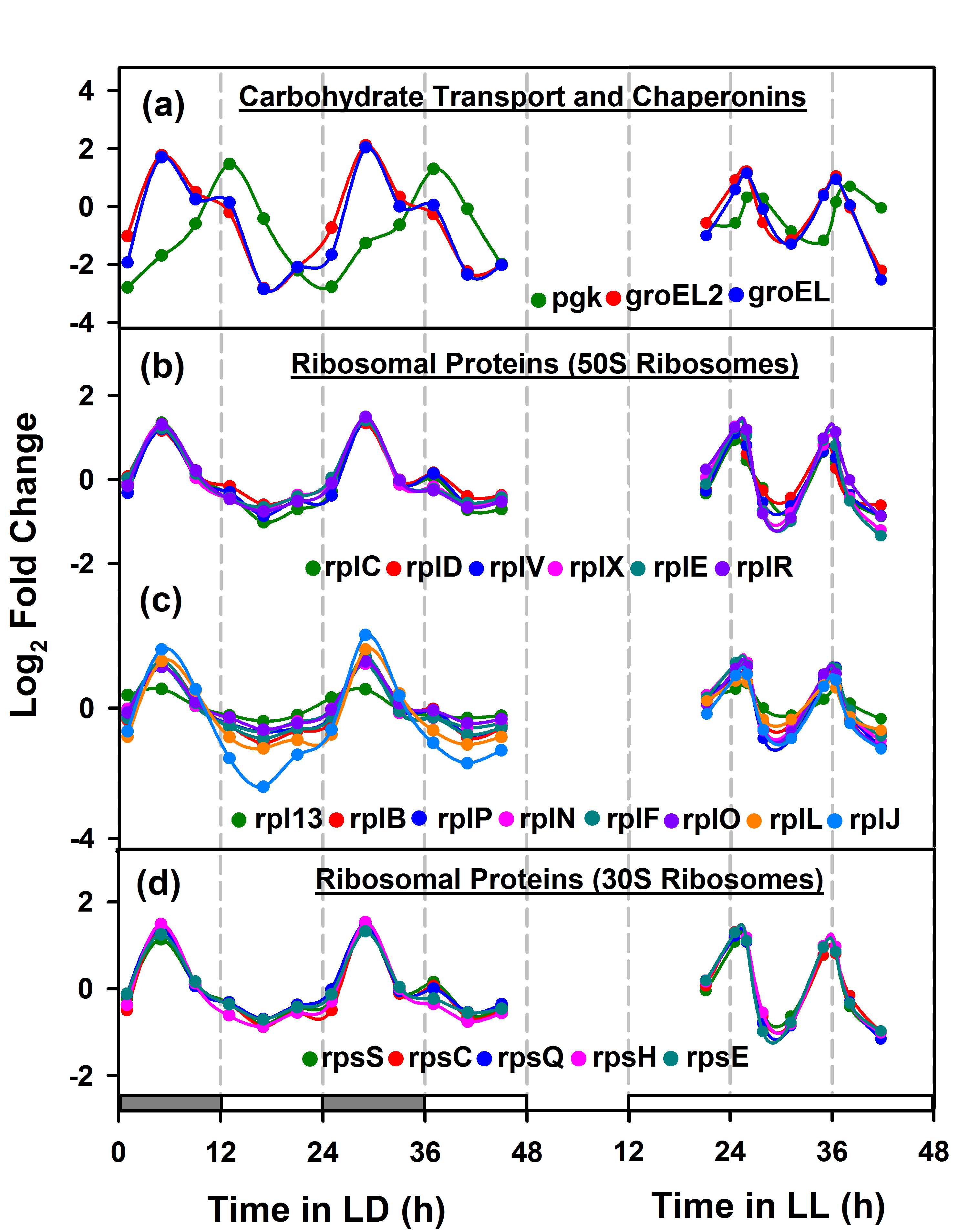

Supplement: S2 Fig — A set of housekeeping genes that reported in Lyngbyamajuscula[53], which show cyclic oscillationsfrom the transcriptomicdata of light/dark (LD) condition[19] and present study (LL) were selected for the analysis. Panel (a-d) shows the oscillation of housekeeping genes, involved in carbohydrate transport, chaperonins and ribosomal proteins. Grey and White filled and continuously white filled boxes in the X-axis, indicate the culture growth conditions of LD and LL, respectively. (TIF) [file pone.0125148.s002.tif]
